# Cyclodextrin inclusion complex inhibits circulating galectin-3 and FGF-7 and affects the reproductive integrity and mobility of Caco-2 cells

**DOI:** 10.1038/s41598-020-74467-1

**Published:** 2020-10-15

**Authors:** Marwan Abdelmahmoud Abdelkarim Maki, Shiau-Chuen Cheah, Omer Bayazeid, Palanirajan Vijayaraj Kumar

**Affiliations:** 1 Faculty of Pharmaceutical Sciences, UCSI University, Taman Connaught, Cheras, 56000 Kuala Lumpur, Malaysia; 2 Faculty of Medicine and Health Sciences, UCSI University, Taman Connaught, Cheras, 56000 Kuala Lumpur, Malaysia; 3 Faculty of Pharmacy, Department of Pharmacognosy, Hacettepe University, 06100 Ankara, Turkey

**Keywords:** Gastrointestinal cancer, Metastasis, Cell death, Cell migration, Carbohydrates, Peptides, Biomaterials, Theory and computation, Translational research, Cancer, Carbohydrates, Immunochemistry, Peptides, Computational science

## Abstract

Galectin-3 (Gal-3) is a carbohydrate-binding protein, that promotes angiogenesis through mediating angiogenic growth factors such as vascular endothelial growth factor (VEGF) and fibroblast growth factor (FGF). There is strong evidence confirming FGF involvement in tumor growth and progression by disrupting cell proliferation and angiogenesis. In this study, we investigated the effect of β-cyclodextrin:everolimus:FGF-7 inclusion complex (Complex) on Caco-2 cell migration, cell motility and colony formation. In addition, we examined the inhibitory effect of the Complex on the circulating proteins; Gal-3 and FGF-7. Swiss Target Prediction concluded that Gal-3 and FGF are possible targets for β-CD. Results of the chemotaxis cell migration assay on Caco-2 cell line revealed that the Complex has higher reduction in cell migration (78.3%) compared to everolimus (EV) alone (58.4%) which is possibly due to the synergistic effect of these molecules when used as a combined treatment. Moreover, the Complex significantly decreased the cell motility in cell scratch assay, less than 10% recovery compared to the control which has ~ 45% recovery. The Complex inhibited colony formation by ~ 75% compared to the control. Moreover, the Complex has the ability to inhibit Gal-3 with minimum inhibitory concentration of 33.46 and 41 for β-CD and EV, respectively. Additionally, β-CD and β-CD:EV were able to bind to FGF-7 and decreased the level of FGF-7 more than 80% in cell supernatant. This confirms Swiss Target Prediction result that predicted β-CD could target FGF. These findings advance the understanding of the biological effects of the Complex which reduced cell migration, cell motility and colony formation and it is possibly due to inhibiting circulating proteins such as; Gal-3 and FGF-7.

## Introduction

One of the most common cancer in the United State is colorectal cancer (CRC)^1^. About 20% of patients which are newly diagnosed with CRC found to have metastatic disease. Moreover, around 30% of early stage CRC patients develop metastatic disease^2^. Blood supply is crucial for tumor growth, solid tumor cannot spread more than 2 mm^3^ in diameter (100–300 cells) without blood circulation. Nevertheless, through angiogenesis tumor have the ability to develop their own blood supply. Angiogenesis starts when tumor cells start secreting angiogenic growth factor to stimulate endothelial cells close to blood vessels, this allow the formation of new vessels to grow towards the tumor. The small tumor has more oxygen and nutrients supply that is able to grow and metastasize to different parts of the body^3^. Recently, inhibiting circulating proteins like galectin-3 (Gal-3), fibroblast growth factor (FGF) and vascular endothelial growth factor (VEGF) have gained interest due to their important role in pathophysiological diseases such as cancer^4,5^. Galectins are carbohydrate-binding proteins that specifically bind to β-galactosides of different types of large glycan^6^. Galectin/glycan complex are able to connect to other galectin/glycan complex forming lattices-like structure on the plasma membrane. This can influence several biological functions among the cells such as cell signaling, cell adherence and cell migration^7^. Gal-3 promotes angiogenesis by numerous mediators such as VEGF and FGF.

Lactose; a Gal-3 inhibitor reduces VEGF and FGF-mediated angiogenesis in vitro which is similar to the effect when Gal-3 is knockdown^8^. Additionally, inhibiting circulating angiogenic factors VEGF and FGF have a beneficial effect by reducing cancer cells survival, migration, and proliferation^2^. FGF and VEGF are angiogenic growth factors that stimulates vascular repair, blood vessel sprouting, and regeneration^9^. FGF and VEGF mediate their effects through binding specifically to their receptors and promote receptor dimerization, this activates receptor intercellular tyrosine kinase domain followed by auto-phosphorylation to allow specific intracellular molecules to propagate the signal from the cell surface. Activation of FGF and VEGF receptors regulate differentiation, proliferation and migration. Molecules that inhibit FGF and VEGF signaling are currently considered to be promising drug-like molecules with some already reached clinical trial^10^. Elevated levels of FGF, VEGF and Gal-3 are detectable in patients with CRC^11,12^.

The Gal-3 and FGF expression correlate with the genesis, progression and development of the malignant tumors^11–13^. In our previous work, we have designed a new class of a potential anticancer complex to enhance the antiproliferative efficacy of everolimus (EV) on Caco-2 cell line. We revealed that the β-cyclodextrin:everolimus:FGF-7 inclusion complex (Complex) improved the anticancer effect of the mammalian target of rapamycin inhibitor EV by enhancing its cellular uptake, intracellular retention, therefore preventing cell proliferation. Also, we showed that the Complex has less toxicity to normal human cell line FHs 74 Int, the selectivity index of the Complex was found to be 18.24^14^. Bioactive molecules can exert a phenotypic effect by targeting one or, frequently, several target proteins. Practically it would be impossible to test each drug-like compound for every possible molecular target. For that reason, computational methods such as structure similarity search and molecular docking can be used to fill this gap and identify the possible targets for each individual molecule. In this study, we investigated the effect of the Complex on Caco-2 cell migration, cell motility, colony formation. In addition, we assessed the ability of the Complex to inhibit circulating proteins; Gal-3 and FGF-7. Structure similarity search and molecular docking were used to identify the molecular target of β-cyclodextrin (β-CD).

## Results

### Target Identification

**Figure 1:**
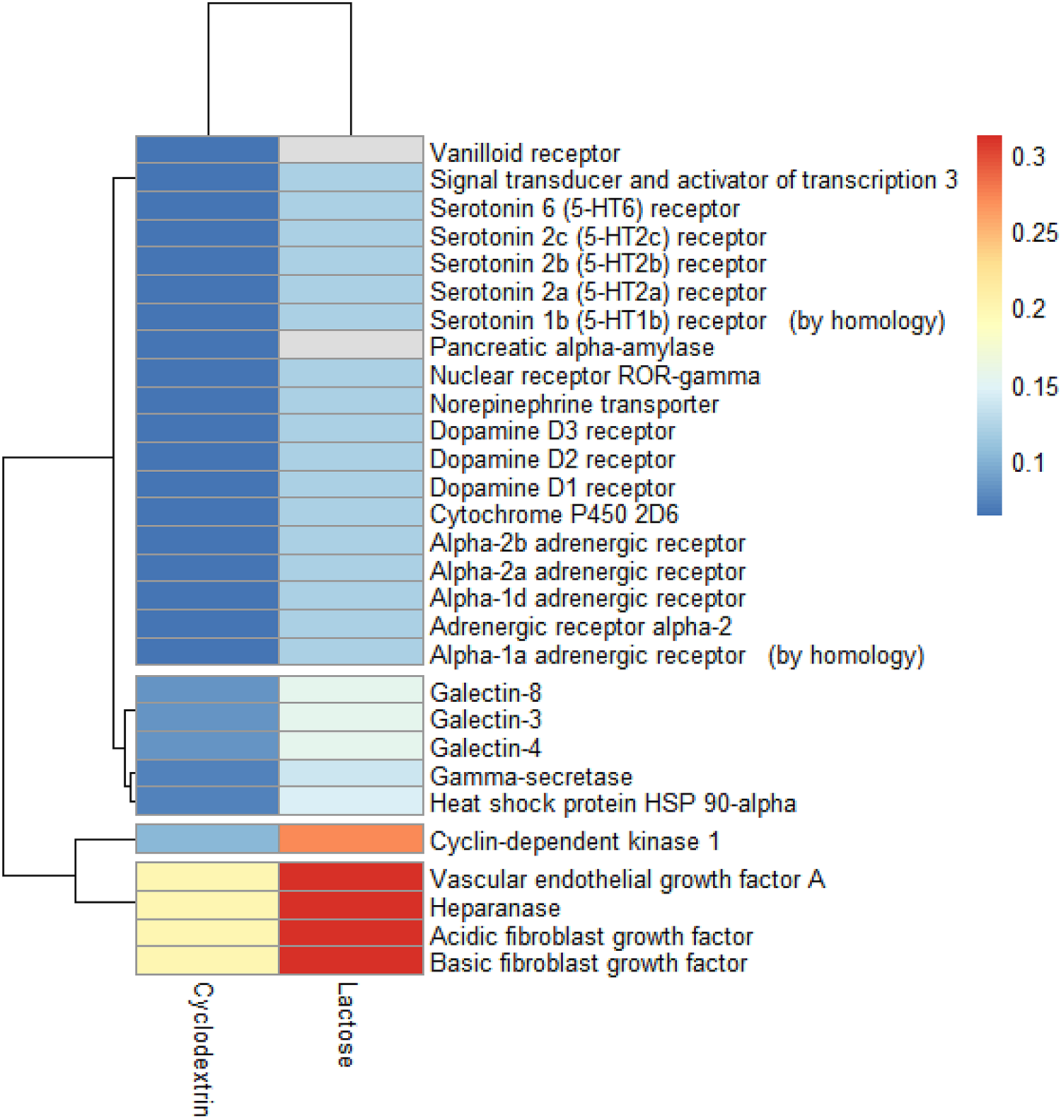
Consensus molecular targets of β-CD and lactose.

Structure similarity search, which is based on the idea that molecule-possessing similar 2D/3D structure may target similar protein targets^15,16^. The obtained results were processed via R Software using (Pheatmap package) (Fig. 1). Swiss predicted that basic FGF and Gal-3 as possible targets for β-CD based on the 2D similarity to CHEMBL198643 (score 0.787) and CHEMBL1669628 (score 0.870), respectively. In comparison, Swiss predicted that lactose (standard galectin-specific sugar) might target basic FGF based on its 3D similarity to CHEMBL198643 with a score of 0.959. In addition, lactose can target Gal-3 based on 2D similarity to CHEMBL1669628 with a score of 0.870. The structure of lactose, β-CD, CHEMBL198643 and CHEMBL1669628 are available in Supplementary Fig. S1.

### Real-time chemotactic cell migration analysis

**Figure 2:**
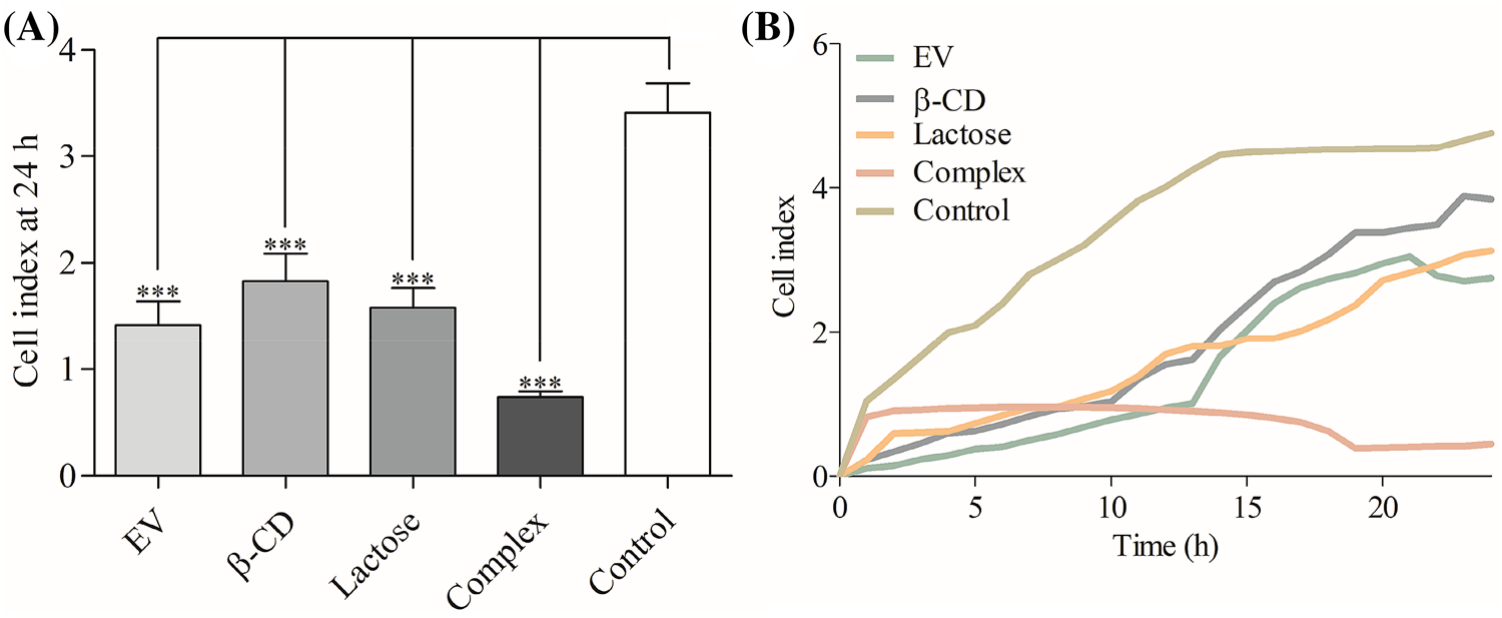
Real-time Caco-2 cells migration was assessed by xCELLigence system. The experiment was performed in triplicate for each treatment and error bars represent $\pm$ S.D. (**A**) Cell index of migrated cells at 24 h after treatments; (**B**) real-time migration of Caco-2 cells, the slop represents the degree of cells migration (cell index) over time. Cells were starved for 24 h before analysis.

The effect of the Complex on metastatic process that dependent on cells migration was investigated. Caco-2 cell line was subjected to a real-time migration assay using xCELLigence system integrated electronically with cell migration (CIM) Boyden chamber (CIM-Plate 16). Over a period of 24 h, starving cells were treated with β-CD, EV, lactose and the Complex. The changes in electrical impedance were recorded as cell index (Fig. 2A), and the slope represents the degree of cells migration over time (Fig. 2B).

### Cell motility measurement by scratch assay

**Figure 3:**
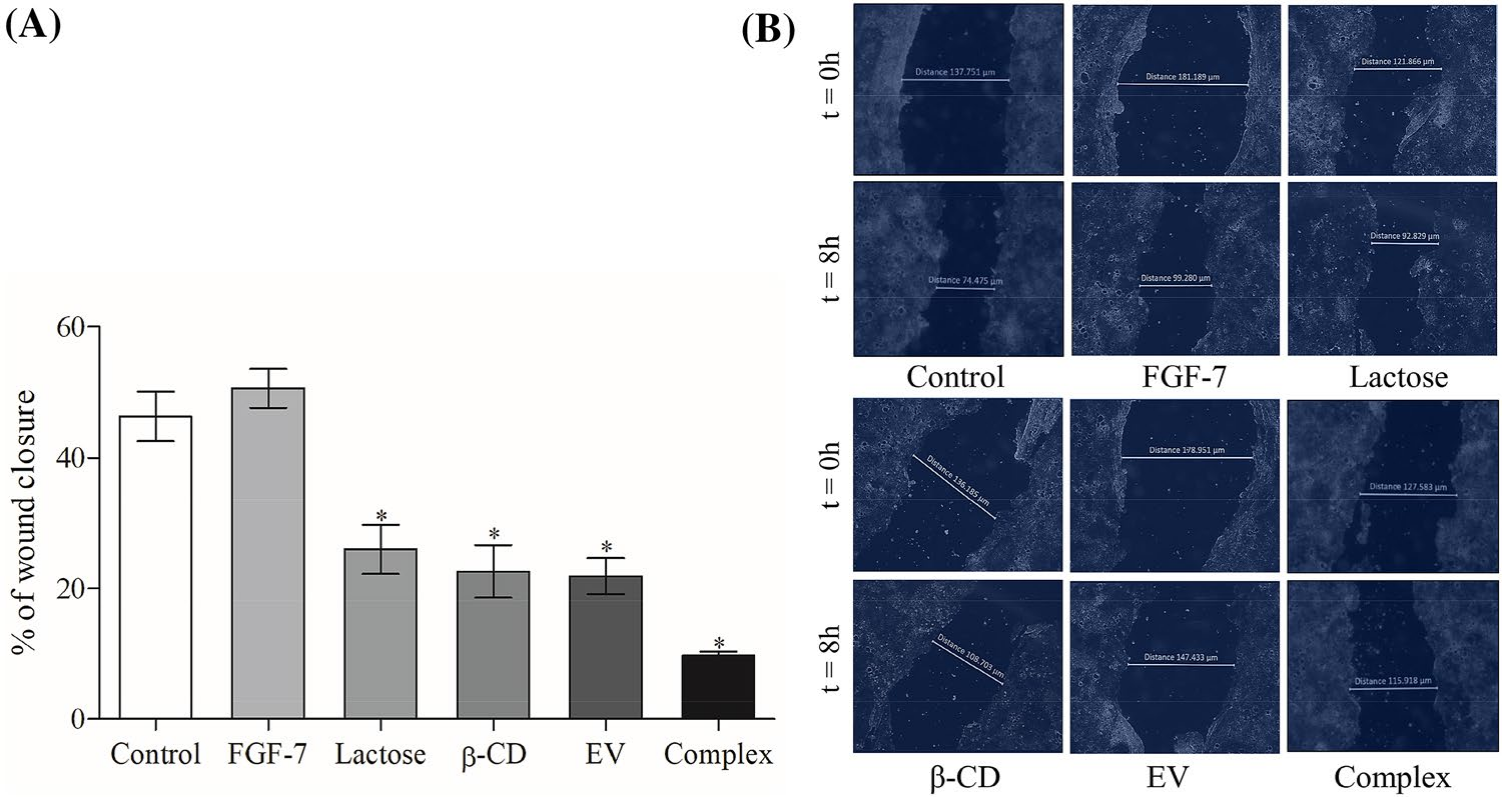
Caco-2 cells motility was tested by scratch assay and assessed by measuring the distance between the boundaries of the migrating cells at 8 h after the treatment (*P < 0.05).

The effect of the lactose, β-CD, EV and the Complex on inhibiting Caco-2 cell motility was investigated by the scratch assay over 8 h. Cell motility was significantly (*P* < 0.05) decreased by the Complex (sub-lethal concentrations treatment) with only ~ 10% recovery compared to the control which has ~ 45% recovery (Fig. 3).

### Colony formation assay

**Figure 4:**
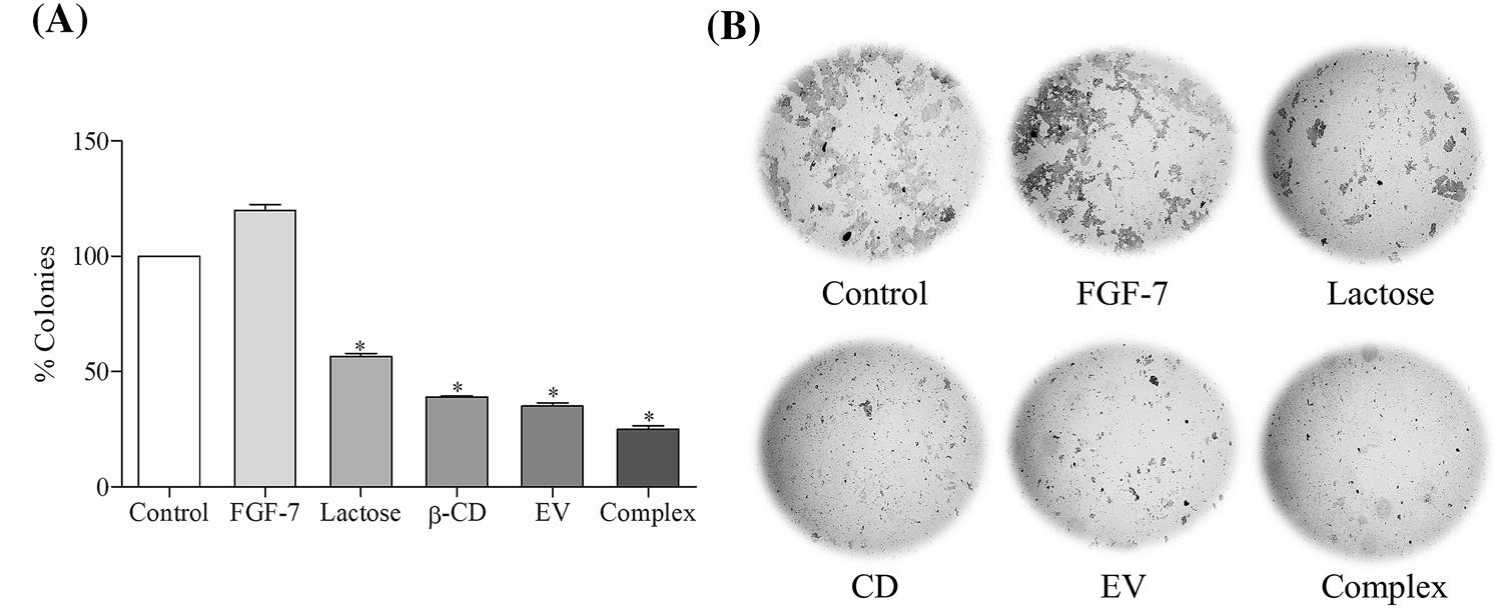
The inhibitory effects of various samples on the colony formation of Caco-2 cells. (**A**) The influence of each treatment on the number of colonies formed, as evaluated by clonogenic assay; (**B**) representative wells of each treatment showing the colony formation after 9 days. Results shown are representative of three independent experiments, control (PBS) versus treated cells (*P < 0.01).

The effect of lactose, β-CD, EV and the Complex on Caco-2 colony formation was measured by clonogenic assay. Figure 4 shows the sub-lethal concentrations treatment of the Complex for 9 days led to ~ 75% inhibition in the growth which is better than the EV (sub-lethal concentrations treatment) alone which has inhibition of ~ 65% (Fig. 4A). The Complex significantly (*P* < 0.01) reduced colony formation compared to the control (Fig. 4B).

### Agglutination inhibition assay

**Figure 5:**
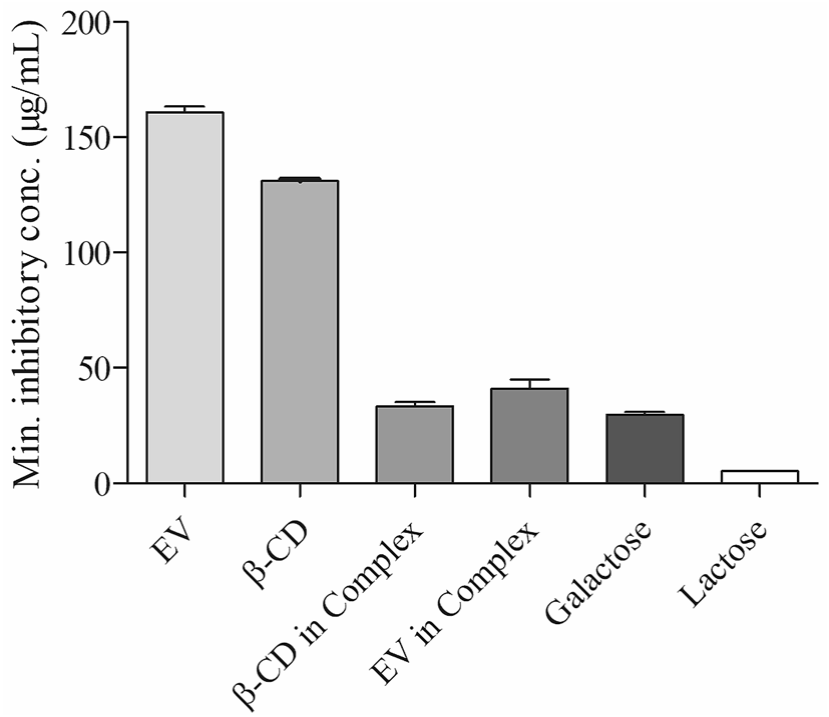
Agglutination inhibitory activity of various samples against the standards galectin-specific sugars (galactose and lactose), values are expressed as mean ± SEM (n = 3).

Hemagglutination inhibition assay was utilized to evaluate Gal-3 inhibitory activity of β-CD, EV and the Complex. The galectin-mediated agglutination inhibition of red blood cells was determined as a minimum inhibitory concentration for each sample. Results were compared with the activity of standards galectin-specific sugars (galactose and lactose). The Complex has the ability to inhibit Gal-3 with minimum inhibitory concentration of 33.46 and 41 for β-CD and EV respectively (Fig. 5).

### Competitive ELISA

**Figure 6:**
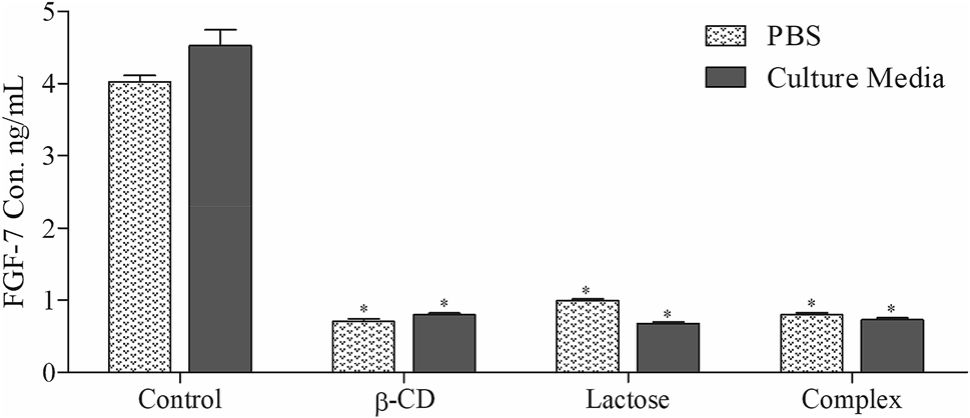
The effect of β-CD, lactose and the Complex on the FGF-7 protein expressing in PBS and Caco-2 cells culture media (cells were treated with β-CD, lactose, and EV:β-CD in culture media containing FGF-7 (4 µg/mL) and incubated for 24 h). The FGF-7 levels in PBS and culture media was measured using FGF-7 ELISA Kit. *FGF-7* fibroblast growth factor 7, *ELISA* enzyme-linked immunosorbent assay (*P < 0.001).

Finally, to invistigate the possible interaction between β-CD and FGF-7, we performed two separate tests: in the first test, FGF-7 (4 µg/mL) only was dissolved in PBS followed by the addition of β-CD, lactose and β-CD:EV and incubated for one hour. Afterwards, the concentration of FGF-7 was measured. In the second test, Caco-2 cells were treated with β-CD, lactose, and EV:β-CD in culture media containing FGF-7 (4 µg/mL). After 24 h, the concentration of FGF-7 was measured in culture media. β-CD, lactose and β-CD-EV interacted with FGF-7 and prevented the antibody to bind to FGF-7 and decreased the level of FGF-7 significantly (*P* < 0.001) both in PBS and culture media. β-CD signifcantly decreased the level of β-CD in PBS and cell supernatant which confirms the structure similarity results that β-CD could target basic FGF (Fig. 6).

## Discussion

Searching for a better delivery system for the known anticancer drug EV (Supplementary Fig. S2), encouraged us to discover and form an inclusion complex (by adding β-CD and FGF-7 to EV) to enhance the antiproliferation effect of EV. In this work, structure similarity search was utilized to identify the possible molecular target of β-CD to shed light on the mechanism behind the Complex activity. Target prediction results revealed that β-CD could possibly target circulating proteins such as; Gal-3 and FGF (Fig. 1). Gal-3 is a carbohydrate-binding protein promotes angiogenesis through mediating angiogenic growth factors such as VEGF and FGF. There is strong evidence confirming FGF involvement in tumor growth and progression by disrupting cell proliferation and angiogenesis. Dalton et al.^17^ have reported that by blocking the carbohydrate recognition domain of Gal-3 with lactose, a significant reduction in cell fusion was observed. Inhibiting Gal-3 could reduce FGF-mediated angiogenesis, which is similar to the effect that associated to Gal-3 knockdown. In our previous work, we reported that β-CD could compete with heparin and prevent heparin from binding to FGF, which inhibits FGF oligomerization and FGF receptor dimerization. This could suppress the intracellular cascades of FGF receptors by preventing its activation and inhibit cell growth^14^. Molecular docking was used to explore the interaction at the binding site between β-CD and the two circulating proteins; Gal-3 and FGF (Supplementary Table S1). Molecular docking insights suggested that β-CD mimicking some of the interaction between lactose (a strong Gal-3 inhibitor) and Gal-3: Glu-184 H-donor, Arg-162 H-acceptor and Trp-181 H-pi (Supplementary Fig. S3). Similarly, β-CD mimicked some of the interactions between the native ligand; heparin tetramer fragment and FGF-7: Lys-130 H-acceptor, Asn-28 H-acceptor, Arg-121 H-acceptor and Lys-130 H-acceptor (Supplementary Fig. S4).

Tumour metastasis is a multistep cascade that usually starts locally with the invasion of the primary tumour into the adjacent tissue, accompany with cancer cells dissemination and secondary tumours formation at distant sites^18^. O'Driscoll et al.^19^ have reported that endogenous Gal-3 could regulate cell migration, since Gal-3 overexpression in lung cancer cell has increased cell motility and invasiveness in vitro. In this study, we measured the effect of EV alone and the Complex on cell migration by chemotaxis cell migration method on Caco-2 cell line. It was observed that EV reduced the cell mobility by 58.47% compared to the control and by adding β-CD and FGF-7 to EV (forming the Complex), they enhanced the effect of EV and decreased the cell mobility by 78.36% compared to the control (Fig. 2). This is possibly due to the synergistic effect of these molecules when used as a combined treatment. According to Dogan et al.^20^, modified rapamycin (as inclusion complex with β-CD or conjugated with polyethylene glycol) outperformed rapamycin in lower concentrations for inhibiting fibroblast proliferation and wound closure of PT-K75 porcine mucosal fibroblasts. In this study, we assessed the effect of the Complex on inhibiting Caco-2 cell motility by using the scratch assay over 8 h. Before conducting the test, sub-lethal concentration of the Complex was tested for its cytotoxic effect and was found to have no effect on the cell proliferation and therefore was used for the scratch assay. The Complex treatment of Caco-2 cells has possessed a significant decrease with ~ 10% recovery compared to the control which has a ~ 45% recovery (Fig. 3). The findings of the scratch assay revealed that the effects of sub-lethal concentration of the Complex on Caco-2 cells is not due to its cytotoxic activity, but simply due to its inhibition of cell motility. Results suggested that the Complex is a possible anti metastatic agent, which supported by its inhibitory effect on cell migration that mentioned previously. To assess the long-term effects of the Complex on Caco-2 cell line, clonogenic assay was used. Gamage et al.^21^ have used the clonogenic assay in vitro to evaluate the tumorigenicity of deoxypodophyllotoxin on Caco-2 cell line. In this work, it was found that colony formation in Caco-2 cells treated with sub-lethal concentration of the Complex over 9 days was suppressed by ~ 75% compared to the control (Fig. 4). These results suggested that the Complex could target the reproductive integrity of cancer cells and prevent clonal expansion.

Hemagglutination inhibition assay was utilized to evaluate the Gal-3 inhibitory activity of the Complex. Stegmayr et al.^22^ used this assay to investigate the inhibition of the Gal-3 canonical galactoside-binding site by the bioactive pectic samples. Gal-3 is known to play a crucial role in events related to metastasis. According to the results presented in Fig. 5, the Complex has the ability to inhibit Gal-3 with minimum inhibitory concentration of 33.46 and 41 for β-CD and EV, respectively. Competitive ELISA test was used to measure the FGF-7 interaction with β-CD, lactose and β-CD-EV both in PBS and culture media containing FGF-7. Results confirmed that β-CD interacted with FGF-7 and decreased the FGF-7 level in PBS and culture media by 82.4% and 82.3%, respectively. Lactose decreased the FGF-7 level by 75.2% in PBS which suggested that lactose possessed weaker interaction to FGF-7 when compared to β-CD. On the other hand, lactose decreased the FGF-7 level in culture media with 10% more than in PBS which could be due to the inhibition of Gal-3 thus inhibiting FGF-7 production in cell supernatant. Similarly, the Complex decreased the FGF-7 level in culture media (83.8%) more than in PBS (80.2%). This indicated that the Complex decreased FGF-7 level in culture media more than in PBS due to the inhibition of Gal-3, therefore inhibiting FGF-7 production (Fig. 6).

## Conclusion

Results revealed that the Complex significantly decreased cell motility, cell migration and colony formation. The Complex treatment has possessed stronger agglutination inhibitory effect compared to β-CD and EV alone. Target identification and molecular modeling insights demonstrated that β-CD could target Gal-3 and FGF-7. The findings of the present work advance the understanding of the biological effects of the Complex which reduced cell migration and motility and it is possibly due to inhibiting circulating proteins such as Gal-3 and FGF-7.

## Materials and methods

### Materials

Caco-2 HTB-37 (ATCC, USA), recombinant human keratinocyte growth factor (rHuKGF) (Sigma Aldrich, USA), β-Cyclodextrin (Sigma Aldrich, USA), everolimus (Toronto Research Chemicals, Canada), β-D-lactose (Sigma Aldrich, Germany), d-(+)-galactose (Sigma Aldrich, Germany), dulbecco’s modified eagle medium (DMEM) (ATCC, USA), trypsin/EDTA (Gibco, USA), streptomycin/penicillin solution (Gibco, USA), fetal bovine serum (Gibco, USA), phosphate-buffered saline (PBS) (Gibco, USA) and KGF (FGF7) ELISA kit (abcam, UK).

### Target identification

Swiss target prediction was utilized to estimate the most probable macromolecular targets of the tested molecules, which were assumed as bioactive. The targets prediction is obtained by the combination of their 2D and 3D similarity with a library of 370,000 known actives on more than 3000 proteins from three different species. SMILES of β-CD and EV were obtained from PubChem and were entered into Swiss target prediction server to predict their molecular targets^16^ and the obtained results were processed via MATLAB Software (MathWorks, Inc., USA). We used R Software for data visualization by using Pheatmap package.

### Real-time chemotactic cell migration analysis

Real-time cell chemotaxis migration of Caco-2 cells was determined by xCELLigence RTCA DP instrument (Acea Biosciences, Inc.). Briefly, 2 $\times {10}^{4}$ cells/well were plated in CIM-Plate 16 (ACEA Biosciences, San Diego, CA, USA). CIM-Plate is containing upper and lower chamber separated with a polyethylene terephthalate membrane with pore size of 8 µm. This membrane contains gold-electrodes on the bottom of the upper chamber to detect the cells movement to the lower chamber. Five percent fetal bovine serum (FBS) was used as chemoattractant and added to the lower chamber. Cell migration and Reattachment rates were recorded quantitatively as Cell Index, a change in electrical impedance of the current flowing through the electrodes. The electrical impedance was recorded every 15 min for 24 h for chemotaxis and every 1–5 min for 2 h for reattachment. Results were presented as a single representative experimental run ± standard deviation (SD) of three wells.

### Cell motility measurement by scratch

Caco-2 cells were seeded (in triplicates) in 24-well plate and incubated under a humidified atmosphere of 5% CO_2_ and 95% air at 37 °C until a confluent monolayer of cells was formed. Uniform scratches were implemented on the cell-monolayer surface using a p-10 pipette tip. Cells were monitored over the course of 8 h while filling the scratched area. Images were taken using light microscope (Axio Vert.A1, Carl Zeiss, Germany). Cell motility was quantified by measuring the distance between the migrating cells boundaries using ImageJ 1.47 software (National Institutes of Health, Bethesda, MD, USA). Cell motility was expressed as a relative percentage of the changes in the distance between the migrating cells boundaries from the start-point to the end-point of each treatment.

### Colony formation assay

Colony forming (or clonogenic) assay was used as a tool to evaluate the effects of novel chemotherapeutic drugs that target the reproductive integrity of cancer cells in a dose-dependent manner as described by Franken et al.^23^. Briefly, Caco-2 cells were seeded in 6-well plate at 1 × 10^3^ cells/well (in triplicates) in 4 mL DMEM supplemented with 3% FBS and with or without treatment and incubated for 9 days in a humidified incubator with 95% air and 5% CO_2_ at 37 °C. Thereafter, cells were fixed with methanol and stained with 0.2% crystal violet (v/v in water). Colonies (≥ 50 cells) were scored using ImageJ 1.47 software (National Institutes of Health, Bethesda, MD, USA).

### Assay agglutination inhibition assay

The evaluation of potential galectin-inhibitors was performed using Microplate agglutination assay according to the protocol that introduced by Nowak et al.^24^. From 10 mL of fresh blood (collected in Alsever’s medium), human erythrocytes were prepared and washed 4 times with 0.15 M NaCl. Four percent erythrocyte suspension was prepared in 0.02 M PBS (pH 7.4), containing 1 mg/mL trypsin, followed by 1 h incubation at 37 °C. The obtained suspension was washed 4 times with 0.15 M NaCl and fixed in 0.02 M PBS (pH 7.4), containing 1% glutaraldehyde, followed by 1-h incubation at room temperature. The fixation process was terminated by adding 0.1 M glycine in PBS (pH 7.4) at 4 °C and the obtained erythrocytes were used for the hemagglutination assay. The assay was performed in a microtitre agglutination assay plate containing DMEM collected from Caco-2 cell culture (the source of Gal-3) in the presence of serially diluted β-CD and the Complex in 0.15 M NaCl. The bioefficacy of β-CD and the Complex as potential galectin-inhibitors was evaluated by determining the Minimum Inhibitory Concentration (MIC) of the tested samples.

### Competitive ELISA

For the binding measurement, ELISA test was used for quantitative determination of rHuKGF using KGF (FGF7) ELISA kit. Briefly, β-CD, lactose and β-CD:EV were diluted at the desired concentration in the sample buffer. rHuKGF (4 µg/mL) was prepared in sample buffer and added to samples well in equal amount. 100 µL of the samples solutions were added to the wells and incubated at 37 °C for 1 h. The wells were washed 5 times with 1 × PBS/0.05% Tween 20 and 100 µL of horseradish peroxidase (HRP)-conjugated secondary antibody and added to each well. After incubation for one hour at 37 °C and washing as above, the bound HRP conjugate was detected by adding 100 µL of tetramethyl benzidine (TMB). The peroxidase reaction was stopped after 15 min by the addition of 50 µL 0.5 M H_2_SO_4_. Optical densities at 450 nm were measured using an ELISA reader. The assay was conducted in triplicates^25^.

### Data analysis

The results are expressed as mean ± SD (n = 6), and the statistical analysis was performed with GraphPad Prism 8 (GraphPad Software, Inc., La Jolla, CA, USA). The significance of any differences between experimental groups was evaluated by the one-way ANOVA followed by a Turkey–Kramer multiple comparisons test. MIC values were expressed as the mean (M) ± S.E.M. (n = 6). The 95% confidence intervals (CIs) of the binding affinity (according to their mean and standard errors) were estimated with 2.5 and 97.5 percentile as the lower and upper bounds. Error bars represent the standard error of the mean.

## Supplementary information

Supplementary Information.
