# Supplementary material for: Cyclodextrin inclusion complex inhibits circulating galectin-3 and FGF-7 and affects the reproductive integrity and mobility of Caco-2 cells
Source: Sci Rep. 2020 Oct 15;10:17468. doi: 10.1038/s41598-020-74467-1 (PMC7562932; doi:10.1038/s41598-020-74467-1)
Supplement: Supplementary file 1 — Supplementary Information. [file 41598_2020_74467_MOESM1_ESM.docx]

**SUPPLEMENTARY INFORMATION**

Cyclodextrin Inclusion Complex Inhibits Circulating Galectin-3 and FGF-7 and Affects the Reproductive Integrity and Mobility of Caco-2 Cells

Marwan Abdelmahmoud Abdelkarim Maki^1^, Shiau-Chuen Cheah^2^, Omer Bayazeid^3^ and Palanirajan Vijayaraj Kumar^1,*^

^1^Faculty of Pharmaceutical Sciences, UCSI University, Taman Connaught, Cheras 56000 Kuala Lumpur, Malaysia

^2^Faculty of Medicine & Health Sciences, UCSI University, Taman Connaught, Cheras 56000 Kuala Lumpur, Malaysia

^3^Faculty of Pharmacy, Department of Pharmacognosy, Hacettepe University, 06100 Ankara, Turkey

*E-mail: vijayarajkumar_p@yahoo.com. Phone: +60103782399. Fax: (+603) 9102 2614.

**
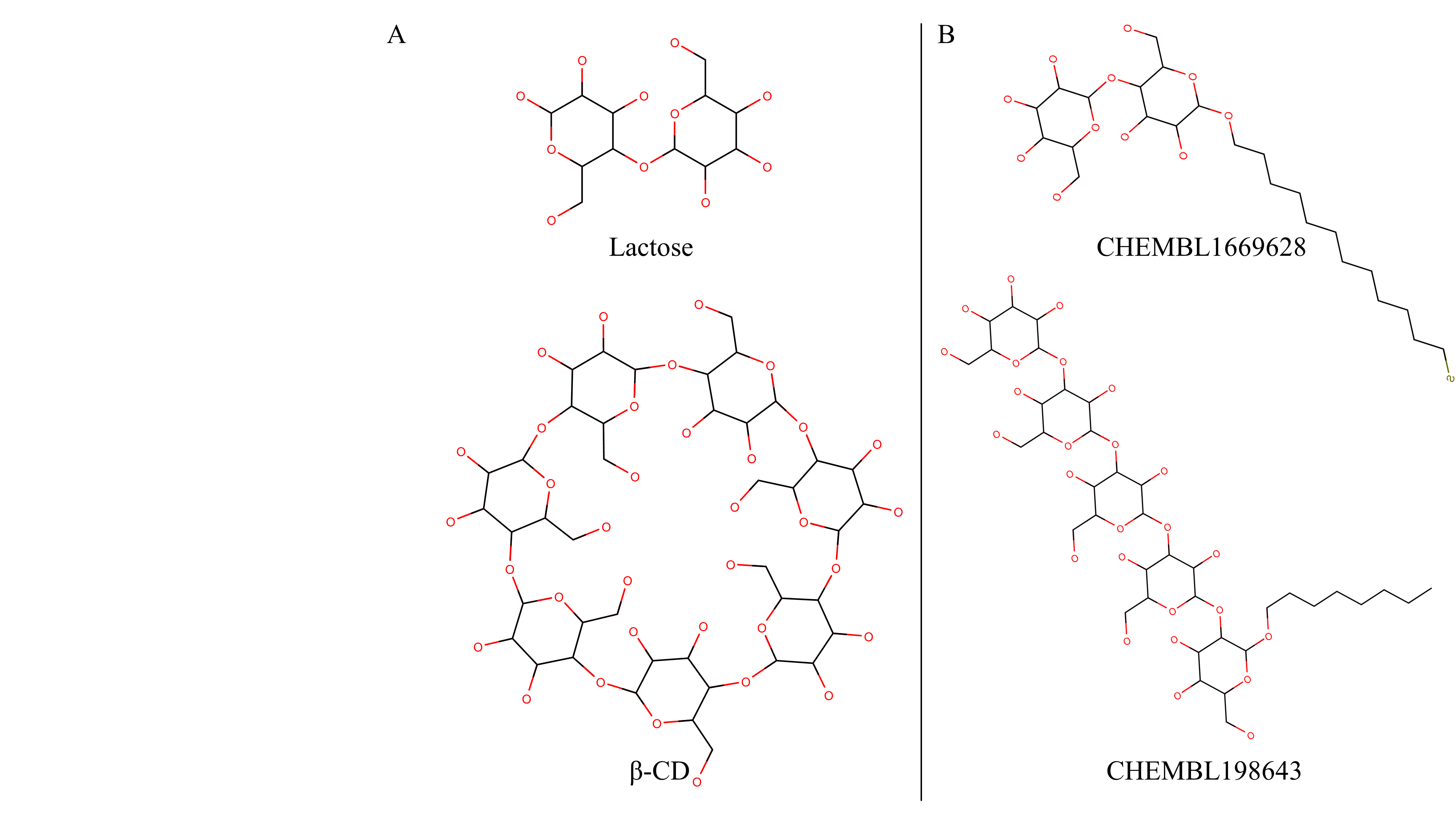
**

**Figure S1.** A) Chemical structure of β-CD and Lactose; B) Chemical structure of the similar molecules; CHEMBL1669628 and CHEMBL198643. Simplified molecular-input line-entry system (SMILES) were downloaded from PubChem and chemical structures were drawn by MarvinSketch 19.17.

**
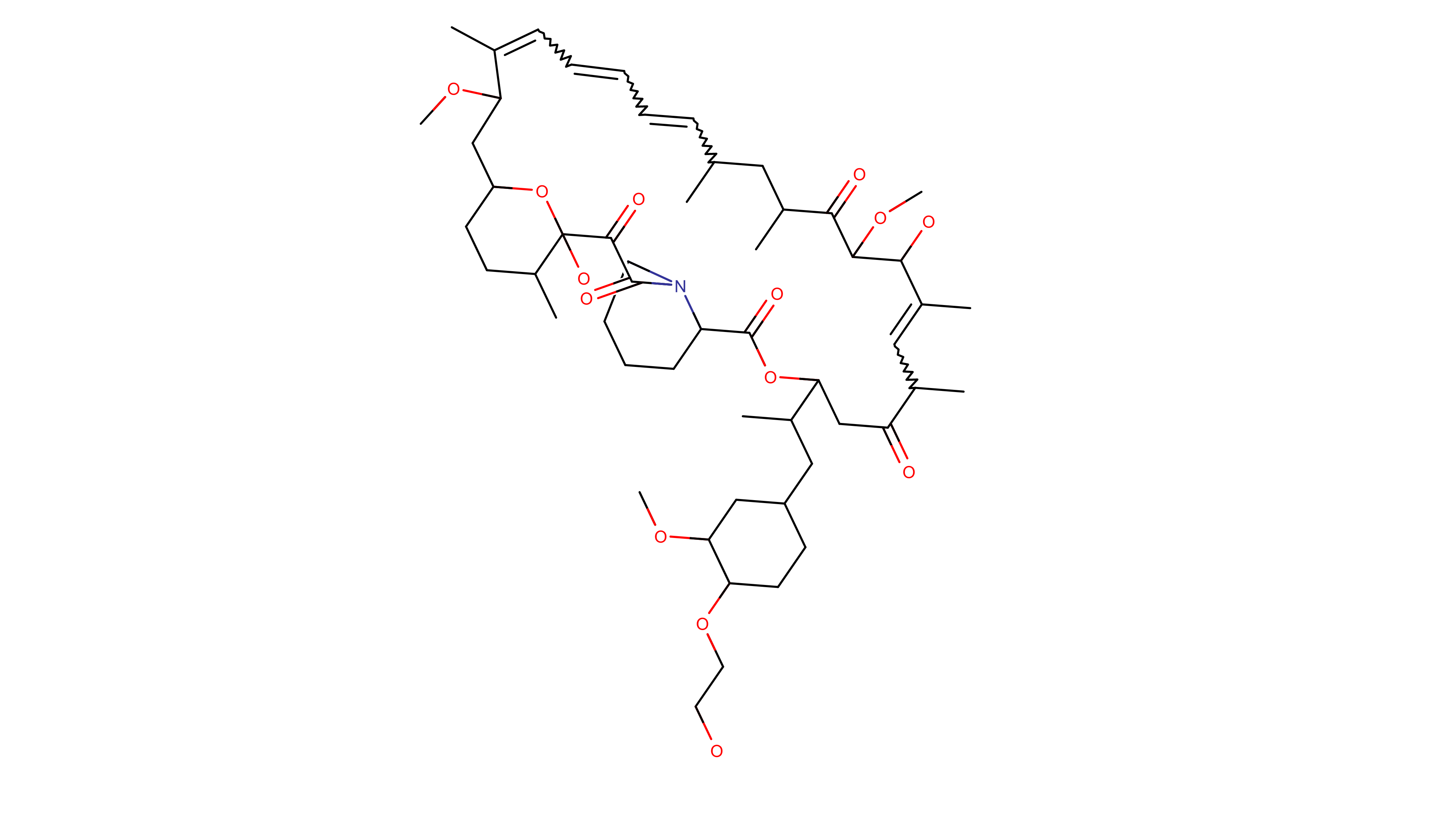
**

**Figure S2.** Chemical structure of Everolimus (EV)**.** EV Simplified molecular-input line-entry system (SMILES) was downloaded from PubChem and EV structure was drawn by MarvinSketch 19.17.

**Molecular Docking**

.Molecular modeling studies were performed to predict the binding affinity of EV and β-CD towards CRD of Gal-3 by utilizing MOE.2014 software (available from the Chemical Computing Group; www. chemcomp.com). The 2D structure of β-CD (PubChem CID: 444041)^1^ was obtained from NCBI PubChem compound database and converted to 3D structure by MOE.2014 software, Chemical Computing Group (CCG; www. chemcomp.com). Molecular docking was performed using MOE.2014 software. The 3D structural complex of FGF7 (PDB ID: 1BFB) and galectin-3 (PDB ID: 3ZSJ) were retrieved from RCSB Protein Data Bank (PDB). All selected small molecules and proteins were optimized and prepared for docking using MOE.2014 software. Proteins retrieved from PDB often have missing atoms. For this reason, correcting the residue library to fix all the issues in the protein followed by 3D protonation. Through Site finder, the binding site was selected, atoms and backbone of the selected site was then isolated. Dummies were also created at the alpha sphere centers. The 3D ligands were minimized in vacuum using MMFF94x with cutoffs of 10 to 12 Å. The hydrogens and charges were fixed, and the RMSD gradient was set to 0.001 kcal/mol. All molecules were added to the database to be docked later. In the docking setting, for initial placement docking; Triangle Matcher London dG scoring was used. For the refinement docking; Forcefield GBVI/WSA dG scoring was used. In all docking, site chain in the binding site were set to Fix (rigid) while ligand set to be flexible. The final docking score put in the account both placement and refinement docking result. RMSD is the ligand location difference between the initial (placement) and refinement.^2–7^

β-CD was successfully docked in the binding pockets of both 3ZSJ and 1BFB. In the binding pocket, hydrogen-bonding and hydrophobic interactions with amino acids were observed. Molecular docking was completed via the molecular operating environment (MOE.2014) with the docking score (London dG - GBVI/WSA dG). Docking scores with the RMSD values are listed in Table S1.

**Table S1**. Docking scores and RMSD values of β-CD in 3ZSJ and 1BFB.

|  | 3ZSJ.pdb | | 1BFB.pdb | |
| --- | --- | --- | --- | --- |
|  | Docking Score | RMSD | Docking Score | RMSD |
| β-CD | -6.173 | 2.207 | -5.931 | 1.543 |

Lactose; a galectin-3 inhibitor which is part of the (3ZSJ.pdb) protein structure was docked into 3ZSJ to optimize the docking setting. The amino acids involved in the binding mode between lactose and 3ZSJ are His-158 H-donor, Glu-184 H-donor, Arg-162 H-acceptor, Asn-174 H-acceptor and Trp-181 H-pi. Visualization the interaction in the binding pocket, docked lactose was able to mimic the interaction of the original lactose (Figure S3-A). β-CD was able to interact with many amino acids in the binding site of 3ZSJ; Asn-143 H-donor, Glu-184 H-donor, Asn-164 H-donor, Arg-144 H-acceptor, Asn-166 H-acceptor, Arg-162 H-acceptor and Trp-181 H-pi (Figure S3-B). Heparin tetramer fragment, which activate FGF-7 was docked into 1BFB to optimize the docking setting (Figure S4-A). β-CD was able to interact with many amino acids in the binding site of 1BFB; Arg-121 H-acceptor, Lys-120 H-acceptor, Asn-28 H-acceptor, Arg-121 H-acceptor and Lys-130 H-acceptor (Figure S4-B).

**
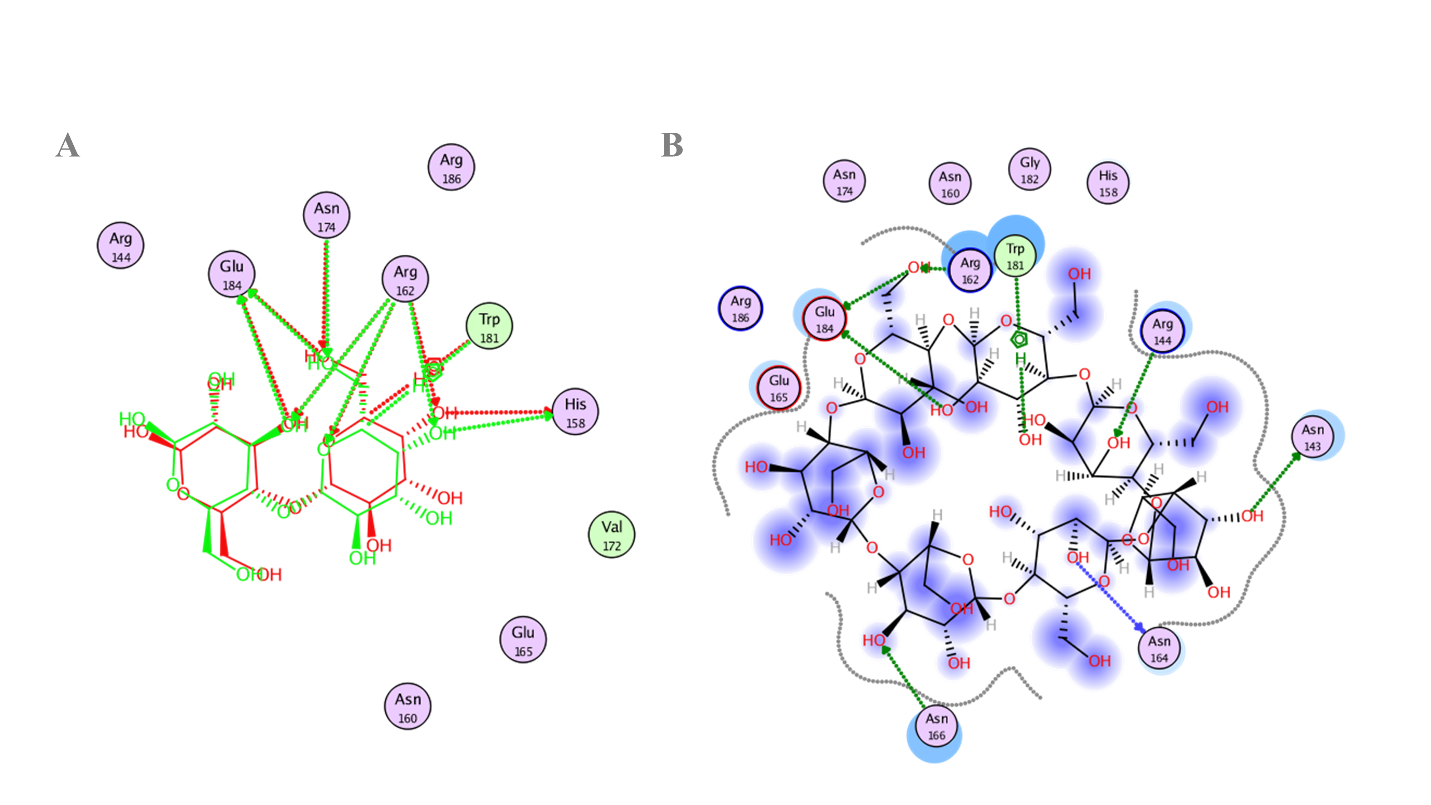
**

**Figure S3.** 2D ligand interaction in the binding site: A) Overlay of lactose in the protein (Red) and the docked lactose (green), B) β-CD in the 3ZSJ binding site.

**
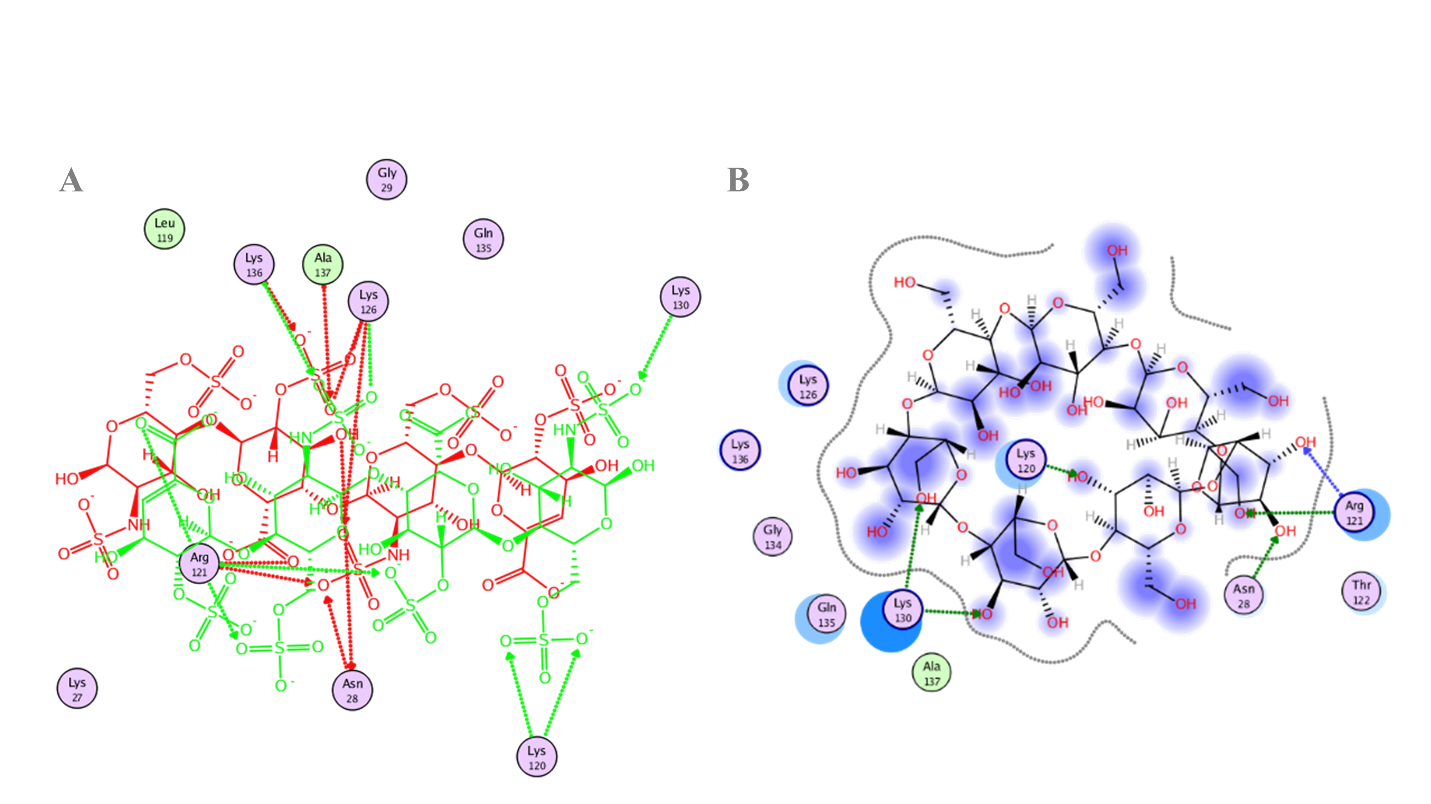
**

**Figure S4.** 2D ligand interaction in the binding site: A) Overlay of heparin tetramer fragment in the protein (Red) and the docked heparin tetramer fragment (green), B) β-CD in the 1BFB binding site.

**Preparation of the Complex**

According to our previous report^8^, the inclusion complex between β-CD and EV was prepared in a 2:1 M ratio. Briefly, EV and β-CD were dissolved in (1:1 v/v) mixture of ethanol and 30% NH_4_OH. The resulted solution was evaporated and dried in a vacuum oven.

**Characterization of the complex**

The size of the complex was measured by Zetasizer and found to be 475.10$\pm$74.83 nm with PdI of 0.605. FGF7:β-CD:EV complex was also characterized by FTIR. All spectra were obtained by a Thermo Fisher Scientific Nicolet iS5 spectrophotometer (Wisconsin, USA) using OMNIC Software from 1000 to 4000 cm^-1^ at a data acquisition rate of 2 cm^-1^ per point. FTIR studies indicate that a new structure was formed and the EV molecule was entirely embedded into the β-CD cavity. In addition, there was a shifting of the broad classic band of β-CD from 3295.22 cm^−1^ to 3287 cm^−1^ indicating the formation of H-bonding between FGF7 and the inclusion complex. The water solubility of the inclusion complex was measured by double beam UV/Vis spectrometer (PerkinElmer, USA). A calibration curve of EV was prepared in ethanol and absorbance was recorded at 267 nm, λmax (n = 6). A known amount of inclusion complex powder was dissolved in distilled water, the absorbance was recorded at 280 nm (λmax of the inclusion complex) and EV concentration was determined (n = 6) accordingly. Furthermore, the FGF7:β-CD:EV complex was prepared by a physical adsorption method (as a result of hydrogen bonding and hydrophobic interaction) by dissolving 6 μg FGF7 and β-CD:EV complex in ultrapure water followed by freeze-drying for over 24 h (at −34°C and gradual ascent up to 20°C), using a Labconco freeze dryer (Labconco, USA) to obtain the FGF7:β-CD:EV complex in a powder form. The aqueous solubility of the inclusion complex was 3.1± 0.23 μM whereas the aqueous solubility of pure EV was 1.7± 0.16 μM. The stability of FGF7:β-CD:EV complex (contains 6.52 μM of EV) in the culture media and the release of EV from the complex was measured by size-restricted dialysis tubing procedure (MWCO 2000 Da, Sigma, Germany). The dissolution medium composed of phosphate buffer solution. The dialysis bag was filled with known amount of complex along with cell culture media, placed in 50 mL phosphate buffer solution, and stirred at 50 rpm under perfect sink conditions. The drug concentration was detected by HPLC (Flexar FX-20, PerkinElmer, USA) at 278 nm. All experiments were carried out in triplicate and performed as at least three independent experiments. According to the data obtained, up to 30 minutes of experiment, there was no detection of free EV from FGF7:β-CD:EV complex sample in receiver compartment, and about 35% of free EV was detected after 1 hour time. This indicated that the complex is stable in cell culture media for at least 30 minutes and exhibits sustain release profile (up to 4 hours)^8^.

**References**

1. β-cyclodextrin PubChem Compound Database. www.pubchem.%0Dncbi.nlm.nih.gov/compound/beta-CYCLODEXTRIN.

2. Hsieh, T.-J. *et al.* Dual thio-digalactoside-binding modes of human galectins as the structural basis for the design of potent and selective inhibitors. *Sci. Rep.* **6**, 1–9 (2016).

3. Seetharaman, J. *et al.* X-ray crystal structure of the human galectin-3 carbohydrate recognition domain at 2.1-Å resolution. *J. Biol. Chem.* **273**, 13047–13052 (1998).

4. Ferreira, L. G., Dos Santos, R. N., Oliva, G. & Andricopulo, A. D. Molecular docking and structure-based drug design strategies. *Molecules* **20**, 13384–13421 (2015).

5. Collins, P. M., Öberg, C. T., Leffler, H., Nilsson, U. J. & Blanchard, H. Taloside Inhibitors of Galectin‐1 and Galectin‐3. *Chem. Biol. Drug Des.* **79**, 339–346 (2012).

6. Diehl, C. *et al.* Protein flexibility and conformational entropy in ligand design targeting the carbohydrate recognition domain of galectin-3. *J. Am. Chem. Soc.* **132**, 14577–14589 (2010).

7. Ritchie, D. W., Kozakov, D. & Vajda, S. Accelerating and focusing protein–protein docking correlations using multi-dimensional rotational FFT generating functions. *Bioinformatics* **24**, 1865–1873 (2008).

8. Maki, M. A. A. *et al.* Molecular Modeling-Based Delivery System Enhances Everolimus-Induced Apoptosis in Caco-2 Cells. *ACS omega* **4**, 8767–8777 (2019).
